# Eukaryotic Expression System *Pichia pastoris* Affects the Lipase Catalytic Properties: A Monolayer Study

**DOI:** 10.1371/journal.pone.0104221

**Published:** 2014-08-18

**Authors:** Madiha Bou Ali, Yassine Ben Ali, Imen Aissa, Youssef Gargouri

**Affiliations:** Laboratory of Biochemistry, National Engineering School of Sfax (ENIS), University of Sfax, Sfax, Tunisia; College of Medicine, University of South Florida, United States of America

## Abstract

Recombinant DNA methods are being widely used to express proteins in both prokaryotic and eukaryotic cells for both fundamental and applied research purposes. Expressed protein must be well characterized to be sure that it retains the same properties as the native one, especially when expressed protein will be used in the pharmaceutical field. In this aim, interfacial and kinetic properties of native, untagged recombinant and tagged recombinant forms of a pancreatic lipase were compared using the monomolecular film technique. Turkey pancreatic lipase (TPL) was chosen as model. A kinetic study on the dependence of the stereoselectivity of these three forms on the surface pressure was performed using three dicaprin isomers spread in the form of monomolecular films at the air-water interface. The heterologous expression and the N-His-tag extension were found to modify the pressure preference and decrease the catalytic hydrolysis rate of three dicaprin isomers. Besides, the heterologous expression was found to change the TPL regioselectivity without affecting its stereospecificity contrary to the N-tag extension which retained that regioselectivity and changed the stereospecificity at high surface pressures. The study of parameters, termed Recombinant expression Effects on Catalysis (REC), N-Tag Effects on Catalysis (TEC), and N-Tag and Recombinant expression Effects on Catalysis (TREC) showed that the heterologous expression effects on the catalytic properties of the TPL were more deleterious than the presence of an N-terminal tag extension.

## Introduction

Lipases (triacylglycerol acylhydrolases, EC 3.1.1.3) catalyze the hydrolysis of triacylglycerols at the interface between the insoluble substrate and water [1] and have been widely used in industrial fields including detergents, dairy products, diagnostics, oil processing and biotransformation due to substrate specificity, regiospecificity, enantiomeric selectivity, thermostability and alkaline stability [2].

Recombinant DNA methods are being widely used to express proteins in both prokaryotic and eukaryotic cells in order to obtain high levels of expression for both fundamental and applied research purposes. For large scale purification of recombinant proteins, affinity chromatography is usually preferred due to the specificity of the process and the relatively easy purification schemes. Chimeric proteins have been made with affinity tags like L-galactosidase, Escherichia colimaltose binding protein, FLAG peptide, glutathione-S-transferase and hexahistidine tag (His-tag) [3]. The His-tag, consisting of four to six consecutive histidine residues, functions as a predominant ligand in immobilized metal affinity chromatography (IMAC) [4]. The ability of the histidine residue complex to bind to metal ions with high affinity even in the presence of denaturing agents and the requirement of mild elution conditions has made the His-tag a versatile tool for protein purification and characterization [5], [6]. However, few comparative kinetic studies have been performed between tagged and un-tagged eukaryotic enzymes.

Recent reports have shown that His-tag can alter the binding characteristics of recombinant proteins when compared to the wild-type native protein [7], [8]. Hang et al. [8] showed that although the His-tagged subunits of the terminase enzyme from bacteriophage (lamda) formed holoenzymes with wild-type catalytic activity, one of the subunits showed altered interaction with DNA. The length, composition and location of His-tag can require further optimization depending upon the sequences of the native protein [7], [9]. Nevertheless, there are several studies where ‘affinity tags’ have no adverse effect on the activity of the native proteins [10], [11], [12].

Recently, Horchani et al [13] studied the interfacial and kinetic properties of the wild type, untagged recombinant and tagged recombinant forms of three staphylococcal lipases (SSL, SXL and SAL3) which were compared using the monomolecular film technique. They showed that independently from the negative effects of the recombinant expression process per se (REC), the presence of an N-terminal tag extension would decrease the catalytic activities (TEC) of staphylococcal lipases by creating a steric hindrance during the interfacial binding step. The effects of the heterologous expression process on the catalytic properties of the staphylococcal lipases are three times more deleterious than the presence of an N-terminal tag extension [13].

No studies have been conducted to compare the kinetic properties of the wild type form of eukaryotic lipases with those of the tagged and untagged recombinant forms.

The turkey pancreatic lipase (TPL) was purified from delipidated pancreases. This avian pancreatic lipase contains 450 amino acids and presents an experimental mass of 49665.31 Da [14], [15]. To investigate the structure-function relationships of this lipase, TPL was expressed in *Pichia pastoris*. The biochemical characterization of the rTPL, using an emulsified system (pH-Stat), and its comparison to the native TPL show that the rTPL seems to be identical to the native enzyme [16].

In this study, we report the expression of the tagged TPL (6His-rTPL) in *Pichia pastoris*. The recombinant tagged enzyme was purified and its kinetic properties are compared to the recombinant and the native forms using the monomolecular film technique in order to study the Recombinant expression Effects on Catalysis (REC), N-Tag Effects on Catalysis (TEC), and N-Tag and Recombinant expression Effects on Catalysis (TREC).

## Materials and Methods

### 1. Lipases

The native turkey pancreatic lipase (nTPL) was purified from delipidated pancreases as described by Sayari et al (2000) [15] and the recombinant TPL was expressed in *Pichia pastoris (X33)* and purified using adapted chromatographic methods, as previously described [16]. Starting with TPL full-length DNA (1353 pb) cloned into the pGAPZαA, which served as the template, the tagged TPL gene was obtained by PCR amplification using the following forward and reverse primers. Forward primer includes 6 Histidine codons (grey) and both primers including an EcoRI restriction site (underlined):

Primer Fw: 5′-GATCGAATTCCATCATCATCATCATCATTCTGAAGTTTGCTATGAC-3′ Primer Rv: 5′-GATCGAATTCTTAGCAAGCAGTAAGGGT-3′

**Figure 1:**
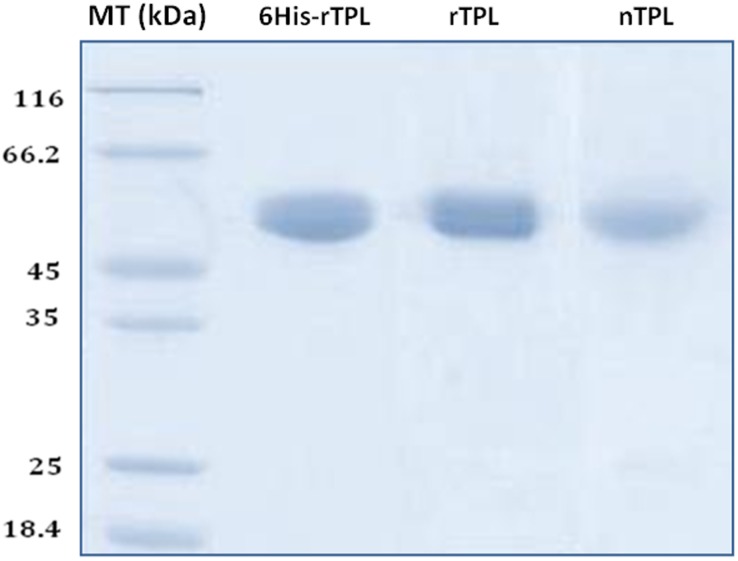
SDS-PAGE of the three TPL forms performed on 12% acrylamide gel. Lane 1: Low molecular weight marker; Lane 2, 3 and 4: purified 6His-rTPL, rTPL and nTPL.

The tagged recombinant TPL was expressed in *Pichia pastoris (X33)* and purified to homogeneity in a single step, using immobilized metal affinity chromatographic method (Figure 1).

### 2. Lipids

1,2-*sn*- and 1,3-*sn*-dicaprin were from Sigma. 2,3-*sn*-dicaprin was prepared from tricaprin (Sigma) by performing stereospecific enzymatic hydrolysis on the *sn*-1 ester bond [17]. As expected, the surface pressure-molecular area curves obtained with 1,2-*sn*-and 2,3-*sn*-dicaprin were found to be superimposable and characteristic of the 1iquid expanded state, and showed no sign of discontinuity throughout the range of surface pressures investigated. The collapse pressure of 1,2-*sn*- and 2,3-*sn*-dicaprin was 40 mN.m^−1^, and that of 1,3-*sn*-dicaprin was 32 mN.m ^−1^ [18].

### 3. Protein concentration determinations

Protein concentration was determined as described by Bradford et al. [19], using BSA as standard. The lipase extinction coefficients were determined using the absorption UV spectroscopy [20]. The protein assays on the three lipases were performed simultaneously and in parallel, using the same chemical reagents.

### 4. Lipase activity and kinetic properties determination

The lipase activity was measured titrimetically at pH 8.5 and 37°C with a pH-Stat using olive oil emulsion or Tributyrin (TC_4_) in presence of 4 mM NaDC and Turkey colipase. One lipase unit corresponds to 1 µmol of fatty acid liberated per min.

The kinetic constants (K_m_app, k_cat_, V_max_, and k_cat_/K_m_app.) calculated from the initial rate activity of native, recombinant and recombinant tagged TPL forms were determined using tributyrine. The effect of TC4 concentration on the hydrolysis rate was measured at various concentrations ranging from 0.68 to 68 mM.

### 5. SDS-PAGE

Analytical polyacrylamide gel electrophoresis of proteins in the presence of sodium dodecyl sulfate (SDS-PAGE) was performed by the method of Laemmli [21]. The proteins were stained with Coomassie brilliant blue.

### 6. Monomolecular film technique for kinetic measurements on lipase

Measurements were performed with KSV-2000 barostat equipment (KSV-Helsinki, Finland). The principle of the method was described previously by Verger and De Haas [22]. It involves the use of a “zero-order” trough with a reaction compartment and a reservoir compartment, which are connected to each other by a small surface channel. The enzyme solution was injected into the subphase of the reaction compartment when the lipid film covered both compartments. A mobile barrier, driven automatically by the barostat, moved back and forth over the reservoir to keep the surface pressure (π) constant, thus compensating for the substrate molecules removed from the film by enzyme hydrolysis. The surface pressure was measured in the reservoir compartment with a Wilhelmy plate (perimeter 3.94 cm) attached to an electromicrobalance, which was connected in turn to a microprocessor programmed to regulate the movement of the mobile barrier. As previously established [22], the sensitivity of the Wilhelmy plate was estimated at 0.1 mN.m^−1^. Two 2-cm magnetic stirrers running at 250 rpm were placed in the reaction compartment. Kinetic measurements were performed at room temperature (25°C). The reaction compartment had an area of 130 cm^2^ and a volume of 130 ml. The reservoir compartment was 148 mm wide and 249 mm long. The monolayers were formed by spreading a chloroform solution at the air/water interface, using a Hamilton microsyringe. Five to ten minutes were then allowed for the chloroform to evaporate and the film to form. Prior to each experiment, the Teflon trough used to form the monomolecular film was cleaned with water and then gently brushed in the presence of distilled ethanol, washed again with tap water, and finally rinsed with double-distilled water.

The aqueous subphase was composed of buffer (10 mM Tris-HCl, pH 8, 150 mM NaCl, 21 mM CaCl_2_, and 1 mM EDTA) prepared with double-distilled water and filtered through a 0.45 µm Milli-pore filter. Any residual surface-active impurities were removed before each assay by sweeping the surface and applying suction. The kinetic recordings were usually linear. Activities were expressed as the number of moles of substrate hydrolyzed per unit time and unit reaction compartment area at an arbitrary lipase concentration of 1 M in the “zero-order” trough.

### 7. Statistical analysis

Experimental results were given as mean value±SD of three parallel measurements. Comparisons between values were analyzed by Student's test for unpaired data, and p<0.05 was considered significant. All statistical analysis were conducted using Microsoft Excel software.

## Results and Discussion

### 1. Variations with surface pressure in the catalytic activities of nTPL, rTPL and 6His-rTPL using the three dicaprin isomers

**Figure 2:**
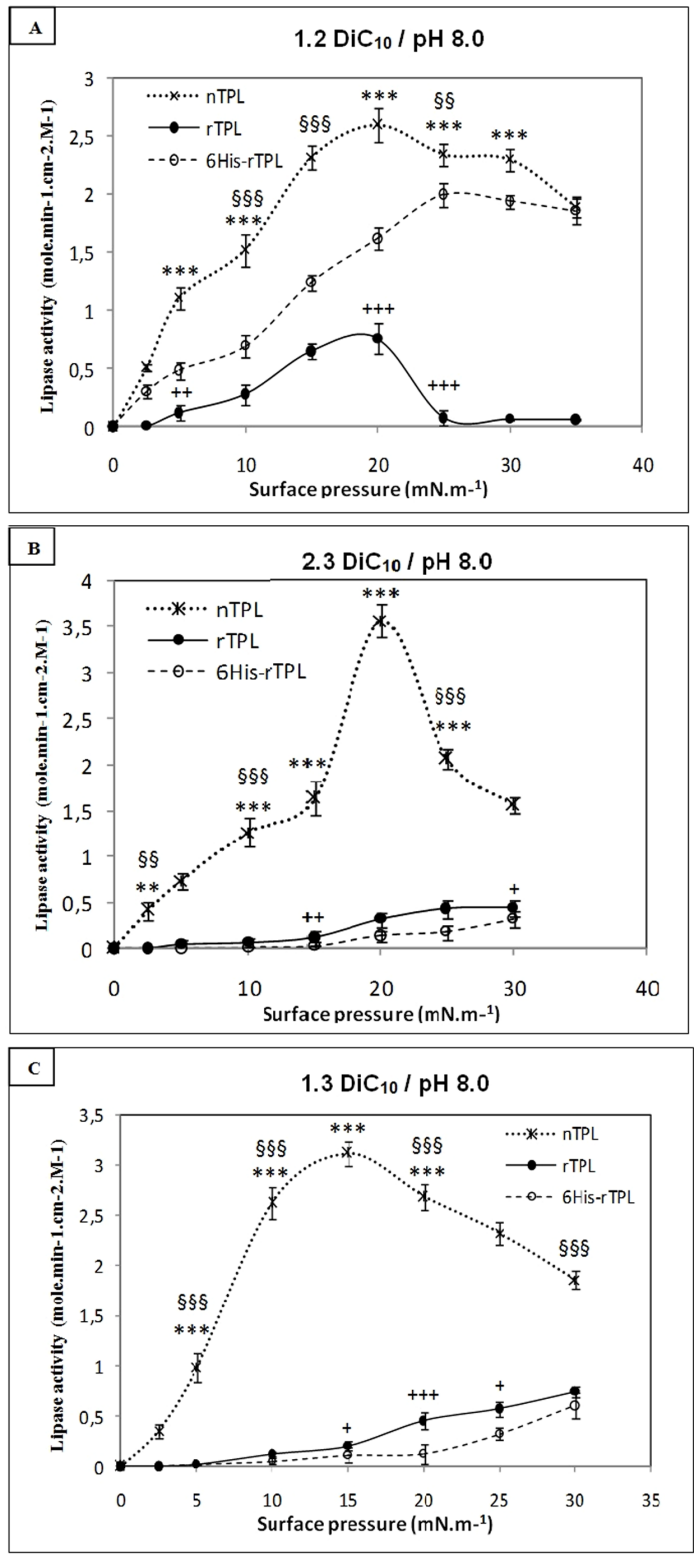
Surface pressure-activity profiles of nTPL, rTPL and 6His-rTPL measured on 1,2-*sn*-dicaprin (A), 2,3-*sn*-dicaprin (B), and 1,3-*sn*-dicaprin (C). Assays were carried out at room temperature in a “zero-order” trough (volume, 130 ml; surface area, 111 cm^2^). Buffer: 10 mM Tris-HCl, pH 8.0, 150 mM NaCl, 21 mM CaCl2, and 1 mM EDTA. Activities are expressed as the number of moles of substrate hydrolyzed by unit time and unit surface of the reaction compartment of the “zero-order” trough for an arbitrary lipase concentration of 1 M. Bars represent means ± SD. *p<0.05, **p<0.01, ***p<0.001; nTPL versus rTPL. **^§^**p<0.05, **^§§^**p<0.01, **^§§§^**p<0.001; nTPL versus 6His-rTPL. **^+^**p<0.05, **^++^**p<0.01, **^+++^**p<0.001; rTPL versus 6His-rTPL.

Kinetic experiments were performed at various surface pressures using three dicaprin isomers as substrates and three TPL forms. As can be seen in figure 2, the three dicaprin isomers were clearly differentiated by all the lipases studied, and this differentiation was more pronounced at high surface pressures.

When using the 1,2-*sn*-dicaprin, the nTPL and rTPL present their maximal activities at a surface pressure of 20 mN.m^−1^, but the 6His-rTPL presents its optimal activity at 25 mN.m^−1^. Up to those pressures, the three lipase form activities decreased (Figure 2A).

**Figure 3:**
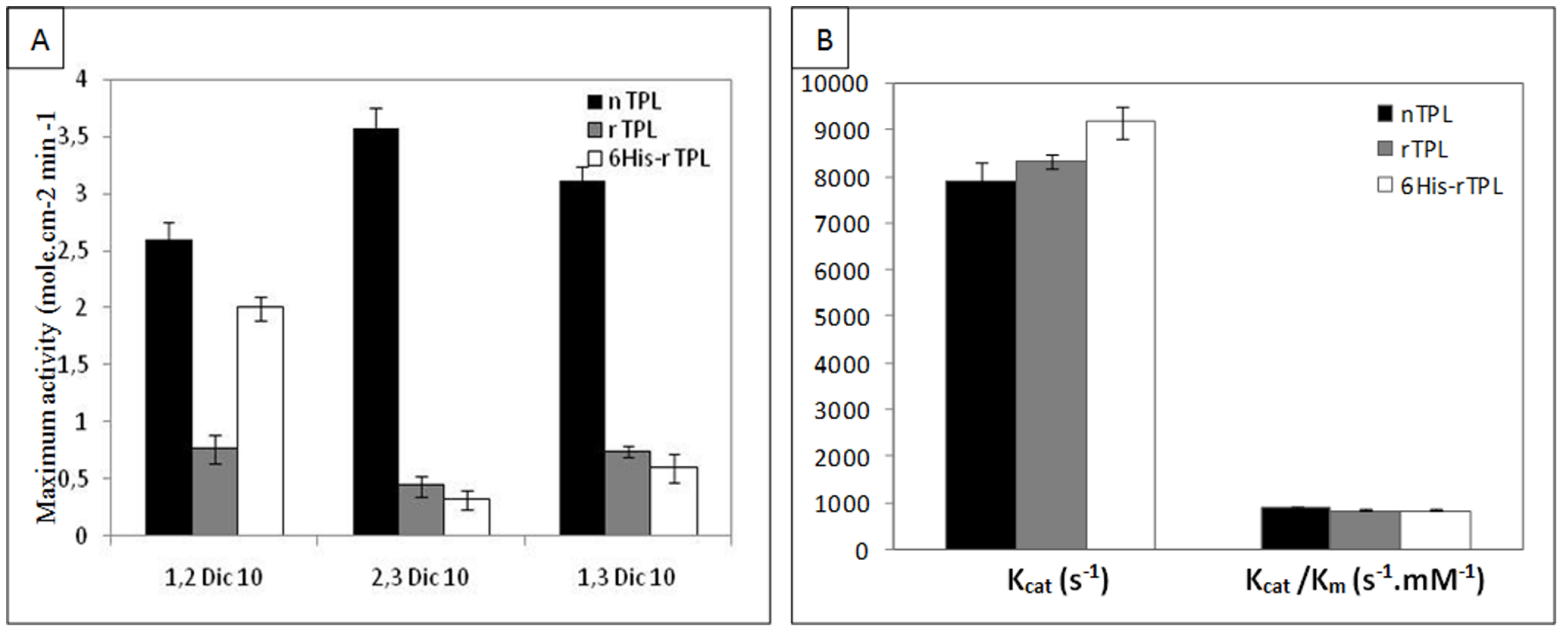
Histogram of the maximum activities of the three TPL forms. (**A**) maximum activities on monomolecular films of 1,2-*sn*-dicaprin, 2,3-*sn*-dicaprin, and 1,3-*sn*-dicaprin, Data based on figure 1. Activities are expressed as the number of moles of substrate hydrolysed per unit time (min) and unit surface (cm^2^) at an arbitrary lipase concentration of 1 M in the aqueous subphase of the reaction compartment of a “zero-order” trough (volume, 130 ml; surface area, 111 cm^2^). (**B**) Histogram of kinetic parameters of TPL forms. nTPL (black bars), rTPL (gray bars) and 6His-rTPL (white bars).

Using 2,3-*sn*-dicaprin and 1,3-*sn*-dicaprin, the nTPL shows a maximum activity with bell-shaped curves at an optimal surface pressure of 20 mN.m^−1^ and 15 mN.m^−1^ respectively, contrary to the rTPL and 6His-rTPL which exhibit increased activity according to pressure showing high pressure preference (Figures 2B and 2C). When analyzing the maximum catalytic activities of the three TPL forms (Figure 3A), one can easily state that nTPL maximum catalytic activities were always higher than those of the rTPL and 6His- rTPL (Figure 3A).

The specific activities of the rTPL and 6His-rTPL are similar when 2,3-*sn*-dicaprin and 1,3-*sn*-dicaprin are used as substrates, but with the 1,2-*sn*-dicaprin, the maximum catalytic activity of the 6His-rTPL is comparable to that of nTPL (Figure 3A).

Our results also show that nTPL hydrolyzes the three dicaprin isomers more efficiently than rTPL and 6His-rTPL do. So, the heterologous expression and N-tag extension change the pressure preference and decrease the specific activity of the native enzyme.

### 2. Effects of recombinant expression and N-tag extension on catalysis (REC, TEC and TREC)

To quantify the effects of the recombinant expression process and/or the presence of an N-terminal extension on the catalytic hydrolysis of the three dicaprin isomers, we coined the terms “Recombinant expression Effects on Catalysis” (REC), “N-Tag Effects on Catalysis” (TEC) and “N-Tag and Recombinant expression Effects on Catalysis” (TREC), which were defined as follows:
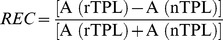


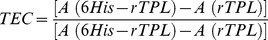


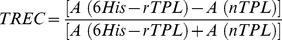
A (nTPL), A (rTPL) and A (6His-rTPL) are the lipase activities of the wild type, untagged recombinant and tagged recombinant TPL, respectively. The TEC, REC and TREC values were calculated from data presented in figure 2 in the case of the three lipases forms. The TEC, REC and TREC panels showed mostly negative values, with all dicaprin isomers and surface pressures tested. The mean REC (−0,7) value showed a deleterious effect of the heterologous expression process on the catalytic properties of TPL in contrast to the presence of an N-terminal tag extension (TEC = +0,03) which did not have negative effects. The combination of the heterologous expression and the N-terminal tag extension (TREC = −0,56) showed that the expression of tagget TPL reduced the negative effect caused by the heterologous expression (Table 1). The positive effect of the N-terminal tag extension was found when 1,2-*sn*-dicaprin was used as substrat, but when 2,3-*sn*-dicaprin and 1,3-*sn*-dicaprin were used, negative effects on catalytic properties of TPL were recorded.

**Table 1:** REC, TEC and TREC values of TPL obtained using 1,2-*sn* dicaprin, 2,3-*sn*-dicaprin and 1,3-*sn*-dicaprin substrates, spread at the air/water interface forming monomolecular films at 20, 25 and 30 mN.m^−1^.

	REC				TEC				TREC			
Pressure (mN.m−1)	1,2-*sn*-dicaprin	2,3-*sn*-dicaprin	1,3-*sn*-dicaprin	Mean^a^	1,2-*sn*-dicaprin	2,3-*sn*-dicaprin	1,3-*sn*-dicaprin	Mean^a^	1,2-*sn*-dicaprin	2,3-*sn*-dicaprin	1,3-*sn*-dicaprin	Mean^a^
20	−0,55	−0,83	−0,72	−0,7	+0,36	−0,38	−0,57	−0,2	−0,24	−0,92	−0,91	−0,69
25	−0,94	−0,66	−0,61	−0,74	+0,93	−0,42	−0,29	+0,07	−0,08	−0,85	−0,76	−0,56
30	−0,95	−0,56	−0,43	−0,65	+0,94	−0,19	−0,1	+0,22	−0,09	−0,68	−0,51	−0,43
Overall mean^b^	−0,7				+0,03				−0,56			

a Mean values of either TEC or REC or TREC for the three dicaprin isomers at a given surface pressure.

b Overall mean TEC, REC and TREC values obtained with the three dicaprin isomers, tested at three surface pressures.

### 3. Regioselectivity and Stereospecificity of TPL

In order to assess the lipase preference for the distal (acyl chains present at sn-1 and sn-3 positions) versus adjacent (acyl chains present at sn-1 and sn-2 or sn-3 and sn-2 positions) ester groups of diglyceride isomers, we have calculated the vicinity index (VI) of native, recombinant and recombinant Tagged TPL as defined by Rogalska et al. 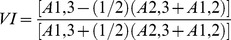, where A1,3, A2,3 and A1,2 are lipases activities measured with 1,3-sn-dicaprin, 2,3-sn-dicaprin and 1,2-sn-dicaprin, respectively. The vicinity index value was calculated at two arbitrary surface pressure values, 15 and 25 mN m^−1^, respectively [18].

**Figure 4:**
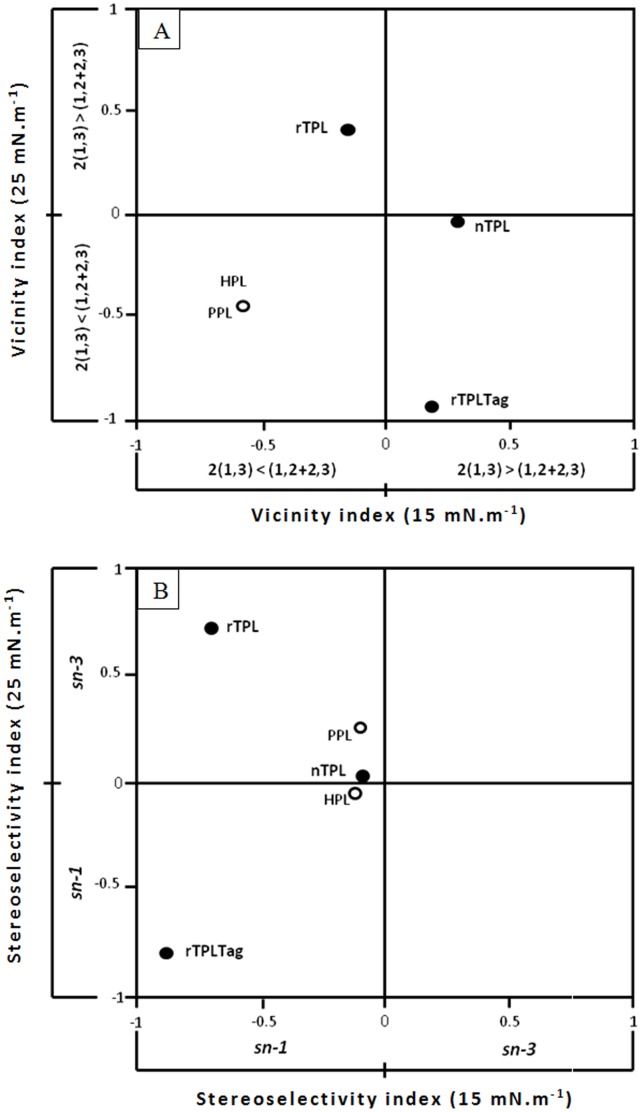
The vicinity and the stereoselectivity index of the three forms of TPL. (**A**) The vicinity index at 25 versus 15 mN m^−1^ is calculated from the specific activities measured at 25 and 15 mN m^−1^, respectively. Data for nTPL, rTPL and 6His-rTPL are derived from figure 1. Data for HPL and PPL are reproduced from previous work [17]. The vicinity index (V.I.) is calculated with the following formula: 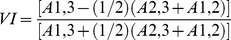, where A1,3, A2,3 and A1,2 are lipase specific activities measured with l,3-*sn*-dicaprin, 2,3-*sn*-dicaprin and 1,2-*sn*-dicaprin, respectively. (**B**) The stereoselectivity index at 25 versus 15 mN m^−1^ is calculated from the specific activities measured at 25 and 15 mN m^−1^, respectively. Data are derived from figure 1. The stereoselectivity index (SI) is calculated with the following formula: 
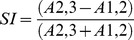
, where A1,2 and A2,3 correspond to lipase specific activities measured with 1,2-*sn*-dicaprin and 2,3-*sn*-dicaprin, respectively.

As shown in figure 4A and table 2, nTPL, as well as 6His-rTPL prefer distal ester groups of the diacylglycerols isomers (1,3-dicaprin) at low surface pressure (15 mN.m^−1^), but at high surface pressure (25 mN.m^−1^) these enzymes prefer adjacent ester of the diacylglycerols isomers (1,2-*sn*-dicaprin and 2,3-*sn*-dicaprin), in contrast to the rTPL which prefers adjacent ester groups at low surface pressure (15 mN.m^−1^) and distal ester groups of the diglyceride isomers (1,3-dicaprin) at high surface pressure (25 mN.m^−1^).

**Table 2:** Regioselectivity and Stereospecificity of nTPL, rTPL and 6His-rTPL.

	V.I.			S.I.		
surface Pressure (mNm^−1^)	nTPL	rTPL	6His-rTPL	nTPL	rTPL	6His-rTPL
**15**	0,248	−0,172	0,202	−0,13	−0,728	−0,97
**25**	−0,033	0,387	−0,98	0,05	0,711	−0,84

The vicinity index (V.I.) and the stereospecificity index (S.I.) of the three TPL forms were calculated using the following definitions: V.I. = [A_1,3_−½ (A_2,3_+A_1,2_)]/[A_1,3_+½ (A_2,3_+A_1,2_)] and S.I. = (A_2,3_−A_1,2_)/(A_2,3_+A_1,2_) at two surface pressure values (15 and 25 mN.m^−1^), where A_1,3_, A_1,2_ and A_2,3_ are lipase activities measured with 1,3-*sn*-dicaprin; 1,2-*sn*-dicaprin and 2,3-*sn*-dicaprin, respectively.

To study the three TPL forms stereopreferences, catalytic activities were measured using 1,2-sn- and 2,3-*sn*- dicaprin at two surface pressure values (15 and 25 mN m^−1^). The stereoselectivity index (SI) was calculated as defined previously by Rogalska et al [18] using the following definition: 
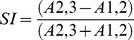
 where A2,3 and A1,2 are lipase activity with 2,3-sn and 1,2-sn-dicaprin, respectively.

S.I. values presented in figure 4B and table 2 show that the three TPL forms are stereospecific for the *sn*-1 position of the 1,2-*sn*-enantiomer of dicaprin at low surface pressures. But at high surface pressures, the 6His-rTPL remains stereospecific for the *sn*-1 position in contrast to the nTPL and rTPL which become stereospecific for the *sn*-3 position of the 2,3-*sn*-dicaprin (Figure 4B; Table 2).

However, the heterologous expression changes the TPL regioselectivity without affecting the stereospecificity contrary to the N-tag extension which retains the TPL regioselectivity and changes the stereospecificity at high surface pressures, so the 6His-rTPL remains *sn*1 specific regardless of the surface pressure. This result can be explained by the large difference between rTPL and 6His-rTPL activities at high surface pressure when 1,2-*sn*-dicaprin was used as substrat (Figure 2A) and the difference between rTPL and 6His-rTPL regioselectivity at high surface pressures where the 6His-rTPL would be specific to the adjacent ester of the diglyceride isomers (Figure 4A).

## General Discussion

Nowadays, *Pichia pastoris* is the most frequently used yeast system for heterologous protein production. Over the last few years, several products based on this platform have gained approval as biopharmaceutical drugs. Protein folding and secretion have been identified as significant bottlenecks in yeast expression systems, pinpointing a major target for strain optimization. However, few studies have investigated the heterologous expression and the N-terminal tag extension effects on the expressed proteins properties.

To our knowledge, this is the first attempt to discriminate between the negative effects of the heterologous expression process and those of the N-terminal tag extension process on pancreatic lipase catalysis. One of the main features of lipolytic enzymes is the fact that, although they are water-soluble, they catalyse substrate hydrolysis at lipid-water interfaces. In the simplest kinetic model developed by Verger and co-workers [22], the coupling between the classical Michaelis-Menten step and various interfacial processes such as the penetration and activation of the lipase and the solubilization of the reaction products were described. It is worth noting that the negative effects of the heterologous expression and N-terminal tag extension processes are not very marked when a partly water-soluble (TC4) and an insoluble (Olive oil) substrates are used (Table 3). When k_cat_ and catalytic efficiency (k_cat_/k_m app_) were calculated, we can see than there are not differences between native, recombinant and recombinant tagged TPL confirming that there are not significant differences between the three TPL forms when classical analysis techniques were used (Figure 3B).

**Table 3:** Specific activity of nTPL, rTPL and 6His-rTPL using olive oil emulsion and TC4 as substrate.

	nTPL	rTPL	6His-rTPL
Specific activity on Olive oil (U/mg)	4300±400	5400±300	6000±300
Specific activity on TC4 (U/mg)	9500±500	10000±200	11000±400

The rTPL and 6His-rTPL seem to share the same biochemical and kinetic properties with the native enzyme under the same experimental conditions (pH stat technique). But when sensitive method such as the mono-molecular film technique was used, we found significant differences in the interfacial properties of the nTPL, rTPL and 6His-rTPL showing the negative effects of the heterologous expression and the N-terminal tag extension on the TPL properties. The most pronounced difference was the significant decrease in the specific activity of the recombinant and the tagged recombinant enzyme in comparison to the native TPL (Figure 3A). We also found that heterologous expression changed the regioselectivity of the TPL without affecting the stereospecificity contrary to the N-tag extension which retained the regioselectivity of the TPL and changed the stereospecificity at high surface pressures. These results show that the recombinant expression and the His tag extension could affect the interfacial properties of expressed lipases.

All in all, expressed lipolytic enzymes must be well characterized to be sure that they retain the same properties as the native ones, especially when they have a therapeutic purpose.
